# Impact of childhood and adolescence cancer on family caregivers: a qualitative analysis of strains, resources and coping behaviours

**DOI:** 10.1186/s40359-023-01406-w

**Published:** 2023-10-28

**Authors:** Adwoa Bemah Boamah Mensah, Humaima Nunoo, Kofi Boamah Mensah, Joshua Okyere, Veronica Millicent Dzomeku, Felix Apiribu, Comfort Asoogo, Joe-Nat Clegg-Lamptey

**Affiliations:** 1 School of Nursing and Midwifery, College of Health Sciences, Private Mail bag, Kwame Nkrumah University of Science and Technology, University Post Office, Kumasi, Ghana; 2 Onwe Government Hospital, Ejusu District, Ejisu, Ghana; 3 Department of Pharmacy Practice, Faculty of Pharmacy and Pharmaceutical Sciences, College of Health Sciences, Kwame Nkrumah University of Science and Technology, University Post Office, Private Mail bag, Kumasi, Ghana; 4 Department of Population and Health, University of Cape Coast, University Post Office, Cape Coast, Ghana; 5 Paediatric Oncology Unit, Komfo Anokye Teaching Hospital, Kumasi, Ghana; 6 Department of Surgery, School of Medical Sciences, University of Ghana, Accra, Ghana

**Keywords:** Paediatrics, Cancer, Caregiving, Qualitative research

## Abstract

**Background:**

The physical demands of caring for children and adolescents diagnosed with cancer, over a lengthy period, exert significant strain on the health and well-being of family caregivers. The capacity of family caregivers to surmount and cope with the various strains they experience due to the diagnosis and treatment trajectory is essential to the quality of life of the child and adolescent who has been diagnosed with cancer. However, the experiences of family caregivers have been under-explored. This study explored the strains, resources, and coping strategies of family caregivers of children and adolescents diagnosed with cancer in Ghana.

**Methods:**

Guided by a descriptive phenomenological design, 20 semi-structured interviews with family caregivers were conducted at a tertiary health facility that provides paediatric oncology services. The study was conducted between June and October 2022. The interviews were transcribed verbatim, translated and coded using NVivo software. An inductive thematic analysis approach using Vaismoradi et al.’s thematic analysis framework was followed in analysing the data.

**Results:**

The study revealed that family caregivers of children diagnosed with cancer experienced three main strains: somatic strains (poor sleep quality, loss of appetite, and unintended weight loss), economic strains (financial burden and loss of economic livelihood), and psychosocial strains (isolation from social activities and network, frustration and helplessness, and balancing multiple family needs). The following themes emerged as coping resources: family cohesiveness, community support, and support from health care providers. Coping strategies that emerged included trusting in God and being self-motivated.

**Conclusion:**

The study concludes that family caregivers experience somatic, economic, and psychosocial strains. However, they can leverage available resources (family cohesiveness, community support, and support from healthcare providers) to cope with these strains. There is a need to educate and sensitize family caregivers about the potential strains that they are likely to experience prior to the assumption of care roles. Also, the formal inclusion of non-governmental organizations and religious bodies will ensure that family caregivers receive sufficient community support to cope with the strains of caregiving.

**Supplementary Information:**

The online version contains supplementary material available at 10.1186/s40359-023-01406-w.

## Background

Paediatric cancer is a health concern that adversely affects the quality of life of children and their family. About 1.8 million children were living with cancer in 2019; of this number, there were 291,319 new cases [1]. Low-and-middle-income countries (LMICs) alone account for about 90% of the global incidence of the paediatric cancer [2]. Available evidence also indicates that about 80% of children diagnosed with cancer are cured in high-income countries due to the availability and accessibility to comprehensive services; however, in LMICs, less than 30% of children diagnosed with cancer get cured [3, 4]. Given the vulnerability of children, family caregivers play a critical role in managing and providing the needs of the child and adolescent who has been diagnosed of cancer.

There is evidence that the physical demands of caring for children with cancer, over a lengthy period, exert significant strains on the health and wellbeing of family caregivers [5]. In Ghana, like many LMICs, the diagnosis and treatment of paediatric cancer are primarily limited to tertiary health facilities; this turns to be in favour of urban dwelling families [6]. The implication of this is that many rural dwelling families that have a child or adolescent diagnosed with cancer have limited accessibility to healthcare. This further means that rural dwelling families must travel long distances to receive treatment for their child/adolescent who is suffering from cancer. The resultant effect of this long-distance travel could be financial strain due to the cost of transportation, separation from the rest of the family, and psychological distress for the family caregiver [6, 7].

It is noteworthy that the capacity of family caregivers to surmount and cope with the various strains that they experience due to the diagnosis and treatment trajectory is essential to the quality of life of the child/adolescent who has been diagnosed with cancer [8]. For instance, one study has shown that parental stress predicts functional outcome in paediatric cancer survivors [9]. Similarly, parental psychopathology has been reported to be significantly associated with psychosocial dysfunction in children diagnosed with cancer [10]. The family adjustment and adaptation response (FAAR) model as postulated by Patterson suggests that “a family balances their demands (cumulative stressors and strains coming from individual, family and community sources) with family capabilities, which include resources (from individual, family and community sources) and coping behaviours” [8]. Thus, highlighting the significance of strains, resources and coping strategies of family caregivers of children diagnosed with cancer.

Despite the foregoing, studies in sub-Saharan Africa, especially Ghana have not explored the strains, resources and coping strategies of family caregivers of children/adolescence diagnosed with cancer. Studies that have attempted to explore this issue have not explored strains, resources and coping strategies together. Resources in this context refers to anything that enables the family caregiver to cope with the numerous somatic, psychosocial and financial strains. For instance, Bekui et al. [6] investigated the psychological and spiritual wellbeing of family caregivers; similarly, Bekui et al. [11] explored the physical and socioeconomic strains that confront family caregivers of children and adolescents diagnosed with cancer. Both studies did not explore the coping resources and coping strategies adopted by family caregivers to overcome the strains they experience. This situation presents a substantial gap in the current knowledge of family caregiving for children diagnosed with cancer in resource-constrained settings like Ghana. To narrow this knowledge gap, the study aims to describe the strains, concerns, resources and coping behaviours of family caregivers of children and adolescents diagnosed with cancer in Ghana.

## Methods

### Study design

To gain deeper insights into the strains of caregiving, a qualitative research approach was followed. Specifically, a descriptive phenomenological design was employed. The authors adopted this study design because it examines and describes the actual experiences of a person rather than what is believed to have occurred during the experience of a particular phenomenon [12, 13]. Given that in this study we are concerned about the actual lived experiences of family caregivers regarding the strains they encounter, and the resources and coping strategies they adopt in executing their caregiving roles, descriptive phenomenology was deemed the most appropriate design.

### Setting

The present study was carried out at the Paediatric Oncology Unit, situated within the Komfo Anokye Teaching Hospital (KATH) in Kumasi, Ashanti Region, Ghana. Established in 1954, KATH is the second largest hospital in the country, boasting a capacity of 1,200 beds and offering comprehensive inpatient and outpatient care across various disciplines [13]. It serves as a primary healthcare provider for patients residing in the central and northern regions of Ghana. Within the Child Health Directorate of KATH, the Paediatric Oncology Unit is situated. This unit attends to patients as young as 15 years old who are diagnosed with cancer, as documented by Dogbe et al. [14]. In the year 2020, the unit diagnosed a total of 112 new cases of paediatric cancer. As one of the two major paediatric cancer treatment facilities in the country, this unit is primarily responsible for managing most childhood cancer cases and acts as a referral centre for neighbouring regions. Hence, the selection of this site was deemed most suitable for the execution of this study.

### Target population

In this study, the target population were primary family caregivers. That is, an individual who assumes the primary responsibility for providing care and support to the child or adolescent who has been diagnosed with cancer [15]. We recognised family caregiver as including a close family member, such as a parent, sibling, or other relative who takes on the role of providing physical, emotional, and often financial assistance to the child or adolescent diagnosed of cancer.

### Sample and recruitment

The study did not have a predefined sample size. Based on a set of inclusion criteria, we used the purposive sample technique to screen a total of 27 family caregivers of children who had been diagnosed with cancer. The inclusion criteria were that the participants must be (a) an adult (i.e., aged 18 years or older); (b) a parent or guardian of the child/adolescent; (c) have a child aged < 18 years diagnosed of any type of cancer (see Table 1); and, (d) a family caregiver to the child/adolescent and be involved in the health decision-making for the child/adolescent. The following criteria were used to exclude participant from the study; (a) caregiver less than 18years and (b) caregiver providing paid service. By employing purposive sampling, the researchers aimed to deliberately select participants who possess relevant experiences and knowledge that could provide rich and meaningful insights into the study [16]. Even though 27 participants were eligible to participate in the study, we interviewed 20 family caregivers. This was because by the 17th interview, no new analytical information was emerging. However, we followed Francis et al.’s [17] stopping criterion by conducting additional three interviews to confirm that we had reached data saturation. The 18th, 19th, and 20th interviews confirmed that we had reached the point of saturation and met the stopping criterion. The participants included mothers (14), fathers (2), grandmothers (2), brother (1), and aunt (1) (see Table 2).

**Table 1:** Background characteristics of the child

FCG’s ID	Age of child	Sex of the child	Diagnosis	Stage at Diagnosis	Duration of Diagnosis
F001	11	Male	Acute Myeloid Leukaemia	High risk	4 months
F002	5	Male	Acute Myeloid Leukaemia	High risk	3 months
F003	5	Male	Wilms Tumour	Stage IV	10 months
F004	5	Male	Burkitts Lymphoma	High risk	4 months
F005	11	Female	Osteosarcoma	Stage II	4 months
F006	9	Male	Wilms Tumour	Stage IV	3 months
F007	7	Female	Rhabdomyosarcoma	Embryonic	4 years
M001	14	Female	Nasopharyngeal Ca	Palliative	3 months
M002	8	Male	T Cell Lymphoblastic Leukaemia	High risk	3 months
M003	5	Male	Acute Lymphoblastic Leukaemia	High risk	3 years
M004	2	Male	Acute Lymphoblastic Leukaemia	High risk	1 year
M005	9	Male	Burkitts Lymphoma	High risk	3 years
M006	4	Female	Acute Lymphoblastic Leukaemia	High risk	5 Months
M007	4	Male	Non-Hodgkins Lymphoma	Stage I	4 months
M008	11	Female	Neuroblastoma	Stage IV	7 months
M009	3	Male	Acute Myeloid Leukaemia	High risk	2 months
M010	9	Male	Neuroblastoma	Stage IV	1 month
M011	12	Female	Rhabdomyosarcoma	Stage IV	1 year, 3 months
M012	5	Male	Rhabdomyosarcoma	Embryonic	1 year, 1 month
M013	3	Female	Germ Cell Tumour	Stage IV	1 year

NB: FCG refers to family caregiver

**Table 2:** Background characteristics of family caregivers

ID	Age of parent	Sex of parent	Marital status	History of cancer in the family	Relationship to child	Health insurance coverage
F001	38	Female	Not married	No	Mother	Yes
F002	70	Female	Married	No	Grandmother	Yes
F003	42	Female	Married	Yes	Mother	Yes
F004	41	Female	Married	Yes	Mother	Yes
F005	39	Female	Married	No	Mother	Yes
F006	26	Male	Not Married	No	Brother	Yes
F007	42	Female	Widow	Yes	Mother	Yes
M001	40	Female	Married	No	Mother	Yes
M002	37	Female	Divorced	No	Mother	Yes
M003	41	Female	Married	No	Mother	Yes
M004	49	Male	Married	No	Father	Yes
M005	32	Female	Married	No	Mother	Yes
M006	36	Female	Married	No	Mother	Yes
M007	36	Female	Married	Yes	Mother	Yes
M008	35	Female	Married	No	Mother	Yes
M009	31	Female	Divorced	No	Aunt	Yes
M010	37	Male	Married	No	Father	Yes
M011	29	Female	Married	No	Mother	Yes
M012	61	Female	Widow	No	Grandmother	Yes
M013	43	Female	Married	No	Mother	Yes

**NB**: Family history refers to whether there has been any other family member who had experienced cancer

### Data collection

Data collection occurred between June and October 2022 using a semi-structured interview guide. HN (second author) collected the data from caregivers through individual, in-depth, face-to-face interviews using a semi-structured interview guide. The in-depth interview allowed the participants to share their views and thoughts on the phenomenon within their setting [18]. The interview guide was structured in four sections. The first section collected data on the background characteristics of both the parent and the child who has been diagnosed of cancer. This was followed by the next section that collected data on the caregivers’ recognition and appraisal of symptoms. The third section of the interview guide explored caregivers’ reaction to the diagnosis of the child or adolescent, while the final aspect explored caregivers’ home experiences with their child’s/adolescents who has been diagnosed of cancer. Details of the questions asked can be seen in Supplementary File 1.

At the point of recruitment of the study participants, we provided an information sheet that detailed the purpose and scope of the study, and other ethical issues. We then obtained a written consent from each of them and scheduled the interview dates. In addition to taking field notes, all the interviews were audio recorded using a tape recorder. Prior to each interview session, we reiterated the purpose, scope and data collection procedures. We also confirmed the written consent obtained by seeking an oral consent. The interviews lasted between 30 and 65 min. The interviews were conducted in Twi, which is the dominant local dialect in the Ashanti region where the study was conducted. Through the interview, participants were encouraged to respond in their own words about their experience with the adolescent/child’s diagnosis, and with sensitive probing and redirection, the interviewer tried to capture details on the phenomenon to enhance full understanding of participants’ realities [18].

### Data analysis

Our data analysis followed an inductive thematic analysis using Vaismoradi et al.’s [19] thematic analysis framework. Vaismoradi et al. [19] outlines a systematic process of familiarisation, code generation, theme development, and refinement to identify and define meaningful themes. To become familiarised with the data, the audio recordings of the interviews were played repeatedly. Following that, we transcribed the audio data verbatim and translated to English language using back-back translation process. To ensure accuracy in interviewing, transcribing, and translation, one of the researchers (ABBM) who is fluent in Twi and English evaluated a random selection of the audio data and transcripts. After the transcription, JO imported the transcripts into QSR NVivo-12 Plus for the coding of the data. Using the ‘code’ function in NVivo-12 Plus, JO assigned codes to the emerging issues from the transcript. Emerging codes were labelled with the participants’ verbatim narratives to retain the intended meaning. The common patterns across the assigned themes were later categorised to constitute themes and sub-themes [19]. The preliminary codes and themes generated were discussed among the research team as a way of refining and coming to a consensus on the final themes and sub-themes.

### Trustworthiness

Qualitative research thrives on rigour and the ability of researchers to ensure the trustworthiness of the study findings [20]. We ensured trustworthiness by ensuring credibility, confirmability, and reflexivity. Confirmability was assured by conducting member-checking with five of the participants a week after the data collection for them to verify the results. None of the participant raised concerns about the content of the transcript. An audit trail of the audio recordings, field notes and transcripts were documented and stored for future confirmatory purposes. For credibility, we carefully recruited participants who met the inclusion criteria. We also transcribed the audio data verbatim to retain the intended meaning of the participants’ narratives. Throughout the data analysis, periodic debriefings were held to agree on the categorisation of codes to form themes and sub-themes. The research team comprised of a trained nurse who is now in academia, oncologists, and a population health scientist. Hence, bringing to the fore diverse experiences that ensured reflexive attitudes. We achieved transferability by providing a thick description of the participants’ characteristics, such as their demographic information, and relationship to the child with cancer. This enables the comparison of the characteristics of the study participants with other populations and assess the potential applicability of the findings to other caregiving contexts.

### Ethical consideration

Ethical approval was granted by the Institutional Review Boards at KATH (KATH IRB/AP/025/22). We anonymised the transcripts before initiating data analysis. Informed consent was obtained from the study participants. This written informed consent was obtained from the participants after had read and understood the purpose, scope and procedures for the conduct of the study, as well as their rights as participants. The availability of a professional psychologist was made known to the participants in case they experienced emotional distress while participating in the study. However, none of the participants needed the services of a psychologist. The audio recordings and transcripts were safely stored and encrypted with a password to prevent unauthorised persons from having access to the data. We followed the Standards for Reporting Qualitative Research [21].

## Results

The result from the thematic analysis is presented under three main categories, namely: strains, coping resources, and coping strategies. Under the category of strains, three main themes emerged: somatic strains (includes poor sleep quality and loss of appetite), economic strains (i.e., financial burden associated cost of child’s treatment and loss of economic livelihood), psychosocial strains (i.e., isolation from social activities and network, frustration and helplessness). The following themes emerged as coping resources: family cohesiveness, community support, and support from health care providers. Coping strategies that emerged included trusting in God and being self-motivated.

### Strains

#### Somatic strains

From the thematic analysis, it was revealed that there were some somatic strains associated with caregiving activities for children who have been diagnosed with and receiving cancer treatment. According to the caregivers who participated in the study, one of the major strains they encountered was a reduction in their sleep quality. Thus, the participants experienced significant dissatisfaction in sleep efficiency, sleep latency, sleep duration, and wake after sleep onset. In their perspective, this poor sleep quality that was experienced could be attributed to the point that they have to intermittently wake up to check how their child is doing. There were times that the fear of losing their child acted as a catalyst for poor sleep quality. The following quotes reflect the experiences of primary caregivers:

*“I sleep less because if the child wakes up, I must also wake up to see the problem at hand. I am tired at every point in time, we travel a lot too. Sometimes, if the child is asleep, I have to wake up to check if there is no bleeding and other stuff like checking how hot she is”* (F005).

*“I barely sleep. Even if I am able to sleep, I would wake up several times. Sometimes, just the fear that I could lose my child to cancer without being awake to see it scares me to wake up. There are also times that the child will be experiencing pains at night, and so I have to wake up to care for him. So, I have not had a good night sleep since my child was diagnosed of this disease”* (M002).

Loss of appetite emerged as another somatic strain that family caregivers experienced. The participants asserted that in the execution of their caregiving role, they often did not feel like eating because they could not bring themselves to eating while their child was experiencing excruciating pains. This is evidenced in the quotes below.

*“At the moment, on my part, I’m not sick, but everything about me has changed because of the difficulty. I sometimes skip meals to keep going”* (F004).

*“Sometimes when I even look at the pains that he’s going through, I lose appetite. It is just recently that I tried eating better”* (M001).

### Economic strains

This theme describes the monetary or financial challenges that were associated with the caring for a child who has been diagnosed of cancer. Mainly, financial burden associated with the cost of the child’s treatment and loss of economic livelihood were the major economic strains experienced by the primary caregiver.

#### Financial burden associated with the cost of child’s treatment

Results from the analysis indicates that while the national health insurance scheme (NHIS) does cover aspects of the child’s treatment, there were many other associated costs that was not covered by the insurance. The participants contended that some of the medications were not a part of the health insurance benefit package. Additionally, some laboratory tests and scan services had to be paid out-of-pocket by the family caregiver and their family. Hence, exacerbating their health expenditure and rapidly depleting their finances. One of the participants had this to say:

*“My main challenge has been the financial aspects, because for every treatment, I have to pay money. I have to buy medicines. I have to take the child for dressing, and I have to pay every single step of the way. Oh, the health insurance covers some of the medications but most of them have been my burden. I have to pay for them”* (F007).

Another participant narrated:

*“We pay for all the lab tests ourselves. It is one of the main bills. I wonder what the insurance card is for because we pay for everything that is required. From ultrasound scans to x-rays, where we sleep, the bed and lab tests, almost everything. On some days, 5 different samples are taken for lab tests. That is how the 4000 cedis [$442] plus on the bill came about”* (M008).

#### Loss of economic livelihood

The study also revealed that some family caregivers lost their source of economic livelihood as a direct and indirect result of their caregiving activities. In the perspective of the participants, they had to quit their current employments to be able to focus on providing undistracted caregiving to their child who had been diagnosed of cancer: *“I got a job as a cook in a new school in the area for two weeks, then this whole issue started to unfold, so I quit. I had to quit the job; I couldn’t go anymore. I had to stop to care for the child”* (F007).

Similarly, there were some participants who lost their jobs and source of economic livelihood not because of a voluntary decision but rather due to limited time to carry out their economic activities. This is what one of the participants had to say: *“I’m into network marketing. We do a lot of talking to people to get clients for my business. That is how I earn my commission to cater for myself and my child. Yet, because of my child’s condition, I don’t have that luxury of time to roam around looking for clients. So, in effect, my business is collapsing”* (M003).

### Psychosocial strains

This theme describes how caregiving for children who have been diagnosed of cancer affects the psychology, emotions, and the social lifestyle of family caregivers. The findings revealed that family caregivers were often isolated from social activities. According to the participants, it was impossible to participate in social gatherings such as festivals, church activities, wedding ceremonies, among others. For instance, one participant had this to say: *“I actually do not go anywhere that I must leave my child at home. I’m almost always at home. But before her condition, I could leave her at home and attend social events with my friends. But now, I cannot do that; I just have to isolate myself because I will not get anyone to my daughter’s needs when I am not around”* (F007).

Another participant also shared that, *“Because of my child’s condition, I cannot participate in normal social activities. I must stay with my child 24/7. That means that wedding, festivals, outings and other social gatherings are a no-go-area for me”* (M013).

Concerning the emotions of the participants, it was revealed that frustration and feelings of helplessness was common among the family caregivers. The uncertainties about the treatment outcome of the child were a factor that exacerbated frustration among the family caregivers. Similarly, the inability to do anything to alleviate the child’s pains often resulted in feeling of helplessness among the family caregivers. For the participants, it seemed like they had been abandoned and left to deal with their financial, emotional and somatic challenges on their own. This is reflected in this quote: *“The stress alone is not easy. I always feel helpless and frustrated. I don’t know what the outcome will be for my child. Besides my wife, there is no other person that I can share this burden with. And so, it is virtually like we are dealing with our own problem by ourselves. It makes me feel totally helpless”* (M004).

The study also revealed that family caregivers struggled to balancing the multiple needs of their families. From the analysis, it is indicative that other members and children of the family required equal attention and support from the caregiver. However, because they unable meet these demands because their priority and attention were skewed towards the child who had cancer. Hence, the needs of other children in the family were significantly affected by the caregiving roles of family caregivers. This assertion is typified in this statement from a mother: *“To be honest, I don’t know what is happening with the rest of my kids and my husband. When we started coming to the hospital, I was trying my best to balance everything, but it was not working for me because the child had to be hospitalised for days, and that meant I had to be away from the family. As to whether they [other children and husband] are feeling well, I cannot tell.”* (M002).

Another family caregiver expressed the view that he had planned to return to school for his higher degree programme and commit some funds towards his building project. However, due to the caregiving role that he assumed, he had to forgo his decision to return to school to be able to effectively deliver their role as a family caregiver. The amount of money that he had to contribute towards the building project had to be reduced to balance the available resources to facilitate the child’s treatment: *“[gasps] I don’t know what to say because balancing my personal needs with the needs of my child has not been easy. I had to abandon my plan to start a postgraduate programme this year. I am also building my own house but now, I cannot spend much money on the project because I must be reasonable and cut off some amount to finance my child’s needs”* (M010).

### Coping resources

The various coping resources available to the family caregivers are described under this theme. The emerging coping resources included family cohesiveness, community support, and support from healthcare providers.

#### Family cohesiveness

Family cohesiveness was one of the major resources that enabled the family caregivers to cope with the associated strains of caregiving. The study showed that families came together in solidarity to support the family caregiver to offset part of the cost of treatment. Some participants asserted that their relatives sold their belongings of economic value to raise sufficient funds to assist the family caregiver to afford the cost of treatment for the child. For instance, one of the participants indicated that *“My sister’s condition actually brought us all together as one family. Although the cost of treatment was high for me, my family came together to assist me. My mother sells yam, my father sells maize and I sell electrical appliances. We all managed to raise enough whenever the need be”* (F006).

Not only did family cohesiveness serve as an important resource to cope with the high health expenditure associated with the child’s treatment. Family cohesiveness also served as a resource that allowed the family caregiver to have someone whom they could rely on for extrinsic motivations in the form of encouragements and goodwill messages. This assertion is evidence in this quote: *“In a way though we are trying to console each other but sometimes men usually don’t like demands. It is not like I am dealing with everything by myself now. I can actually rely on my husband and my extended family for encouragement to persevere and do the best for my chid even amidst these circumstances. It has really been instrumental in keeping my hopes alive and morale high for my child”* (M006).

#### Community support

The participants noted that they received support from different sections of the community to help them cope with the strains associated with caregiving for their children who have been diagnosed of cancer. These supports from the community mainly came from significant agencies and agents such as the religious entities and non-governmental organisations (NGO). Religious entities such as the church provided spiritual and psychological support by praying for the caregivers and their families, as well as using scripture to encourage them to not give up on their caregiving activities for the child. This is exemplified in this quote: *“My church pastor’s support is mostly about praying with me, consoling me and telling me never to lose hope. Essentially, my church has been an instrumental agent in helping me to cope”* (F005).

NGOs served as an essential resource for the caregiver through their donations that helped them to cope with the economic strains. Some NGOs provided funds that contributed to reducing the amount of money that the family caregivers would ordinarily have to pay in full: *“Sometimes, we are lucky. Some of the NGOs come around and donate some amount which helps me to manage the high cost of healthcare for my child by cutting down our bills”* (M009).

#### Support from healthcare providers

Another resource that facilitated the coping behaviours of family caregivers was the support that they received from the healthcare providers. Healthcare providers treated family caregivers with respect and dignity. This behaviour from the healthcare providers made the family caregivers to feel comfortable to share their challenges with them. According to the participants, this friendly behaviour of the healthcare providers was relevant in helping family caregivers to maintain a health state of mind: *“The doctors and nurses are very caring and friendly and make us feel at home. Because of that, I was able to share my deepest fears and concerns with them, and that helped me to calm down and deal with my anxieties. They [the healthcare providers] don’t know this; but their kind words and friendliness are a powerful tool that allows parents like me to get through the challenge”* (F003).

Results from the analysis also showed that some healthcare providers provided family caregivers with money to help them manage the high cost associated with travelling to the health facility for treatment, as well as to reduce the amount of money that they would have paid for laboratory tests. A participant shared this sentiment: *“The nurses have been very supportive in helping me to cope with my financial costs associated with my child’s healthcare. When they [health providers] have money on themselves and you are due to do some laboratory tests and chemotherapy, they sometimes pay for me. They sometimes buy some of the medicines for me and even pay my lorry fare”* (F005).

### Coping strategies

Family caregivers of children who have been diagnosed of cancer adopt certain strategies and behaviours to cope with the various strains that they experience. This theme describes the specific coping strategies adopted by family caregivers. The emerging coping strategies included trusting in God and being self-motivated.

#### Trusting in God

Trusting or believing in the providence of God was one of the common coping strategies adopted by all the family caregivers who participated in the study. Ghana is a nation dominated by Christians; the participants in this study mainly belonged to Christianity. Hence, the family caregivers relied on stories and characters from the bible who had faced certain traumatic experiences to encourage themselves. These stories restored their shattered hopes, and provided some level of assurance that just as it was with the biblical characters, their moment of relief would eventually come. This assertion is shown the quotes below:

*“I hold on to my faith and use some scriptures in the Bible and Bible characters like Job and Joseph to encourage myself and made me believe more that it’s just a test of faith. My latter end will be glorious. Looking at how God helped the other child’s condition to be better, I’ve just put my trust in Him and know that I will be able to go through this phase in my life”* (F003).

*“Temptations will meet us on the way as Christians. You just have to have the faith to overcome such circumstances. There will always be issues with your kids, you should be able to adjust to suite the current situation. I just liken my child’s condition to the suffering of Job in the bible. That trust I have in my God is what has kept me going”* (F005).

#### Being self-motivated

Some of the participants noted that the strains that they experienced during their caregiving propelled them to adopt active coping strategies like being self-motivated. In the view of the participants, there was no one to provide support to them. Therefore, it was necessary for them to develop an intrinsic motivation to persevere through their challenges. The following quotes reflect this theme:

*“I have developed the habit of encouraging myself in this life because nobody cares enough to help. Every day, I tell myself that one day this will pass away”* (F004).

*“I just encourage myself to move on. There is nothing serious that can do to change my situation. And so, I try to gather strength to go through the psychological trauma and economic challenges”* (M004).

## Discussion

Although there are previous studies from Ghana that have discussed the effects of the caregiving on family caregivers of children with cancer, the present study provider deeper insights into the strains, coping resources and coping behaviours of family caregivers of children diagnosed with cancer in Ghana. Poor sleep quality and loss of appetite were the main somatic strains that was experienced by family caregivers of children with cancer. This result is corroborated by earlier studies [8, 11, 22]. Perhaps, the poor sleep quality and loss of appetite experienced by the family caregivers could be explained by the high level of stress and burnout that they experience in executing their caregiving duties. If such patterns in sleep quality and eating pattern continues, it is likely to result in the development of adverse health effects (e.g., obesity, musculoskeletal disorders, and hypertension) among the family caregivers [23]. Therefore, there is a need for interventions that will attenuate the somatic strains experienced by family caregivers.

Consistent with previous studies [11, 24, 25], the study found that family caregivers encounter economic challenges during the conduct of care roles. Many of the participants had to quit their jobs to be able to effectively care for their child. This result aligns with Patterson et al.’s [8] study that found economic strains to be among the major impacts of caregiving for children with cancer. Possibly, the findings can be explained from the point that “the trajectory of childhood cancer care is physically demanding; involving long duration, spanning from hours to weeks, months and years” [11]. Consequently, it demands the family caregiver to be physically present, escorting the child for laboratory test, reviews and treatment. This demand to be physically present exacerbates the likelihood for quit their jobs to care for their child on a full-time basis. Also, the high health expenditure and transportation cost associated with the child’s treatment also worsened the financial wellbeing of the family caregivers. This is synonymous to Renner et al. [24] and Santacroce et al. [25] study. Like many low-and-middle-income countries, there are two stages to the treatment of paediatric cancer in Ghana: the initial, hospital-based intense phase, and the subsequent phase which include continuous follow-up visits till recovery [26]. These phases require the family caregiver to travel to the health facility. This implies that transportation-related costs will be incurred which are not covered by the national health insurance scheme.

The study also revealed that family caregivers of children with cancer in Ghana experienced psychosocial strains. Specifically, the family caregivers intentionally isolated themselves from social gathering activities to have sufficient time to care for their children. Similar findings have been reported by Bekui et al. [11]. Social isolation has significant implications on the psychological wellbeing of family caregivers [27]. In this study, the participants asserted that they felt frustrated and helpless as a result of social isolation. Thus, the results call for a conscious effort to enhance the social wellbeing of family caregivers. This could be achieved through the implementation of health education sessions for family caregivers when they accompany their children for treatment. Perhaps, family caregivers can be encouraged to join social groups that constitute other individuals whose children have been diagnosed with cancer.

Notwithstanding the strains that confronted the caregiving experience, family caregivers exploited the available resources, namely: family cohesiveness, community support, and support from healthcare providers. This result is consistent with Patterson et al. [8] study that showed that family caregivers utilised several resources including family cohesiveness, community support, and support from healthcare providers to cope with the strains of caregiving. The study revealed that NGOs and religious organisations were important resources whose contributions helped family caregivers to cope with the financial and psychosocial strains of caregiving. This observation aligns with a related study from India [28] that revealed that NGOs play an essential role as a resource that allows family caregivers to cope with the strains of caregiving. Bekui et al. [11] and Toledano-Toledano et al. [29] study also corroborates the result that family cohesion and religious support are critical resources that create the opportunity for family caregivers to cope with the strains of executing their care roles.

Consistent with evidence from previous studies [6, 30], this study emphasised the significance of religious or spiritual beliefs as a coping strategy for family caregivers. Probably, the findings can be explained from the point that family caregivers’ trust in God restores their shattered hopes, and provides security, solace, sociability, and self-absolution [31]. The study also suggests that family caregivers draw from their past experiences to intrinsically motivate themselves to cope with the somatic, psychosocial and economic strains that they encounter.

### Strengths and limitations

This study was limited to only family caregivers of children/adolescence with cancer who are surviving. As such, the findings do not provide insight about the strains, coping resources and coping strategies adopted my family caregivers of children with cancer who eventually died during the treatment trajectory. Nevertheless, the study’s strength lies in the use of an appropriate qualitative methodology. Also, the authors ensured reflexivity to limit the likely occurrence of researcher bias.

## Conclusion

The study concludes that family caregivers experience somatic, economic and psychosocial strains. However, they can leverage available resources (family cohesiveness, community support, and support from health care providers) to cope with these strains. There is a need to educate and sensitise family caregivers about the potential strains that they are likely to experience prior to the assumption of care roles. Also, the formal inclusion of non-governmental organisations and religious bodies will ensure that family caregivers receive sufficient community support to cope with the strains of caregiving.

### Electronic supplementary material

Below is the link to the electronic supplementary material.

Supplementary Material 1

## Data Availability

The data are not publicly available due to the ethical obligation to protect the privacy and anonymity of the research participants. However, the data used to support the findings of this study are available from the corresponding author (ABBM) upon reasonable request.

## List of abbreviations

FAAR: Family adjustment and adaptation response
KATH: Komfo Anokye Teaching Hospital
LMICs: Low-and-middle-income countries
