# Supplementary material for: Impact of childhood and adolescence cancer on family caregivers: a qualitative analysis of strains, resources and coping behaviours
Source: BMC Psychol. 2023 Oct 28;11:361. doi: 10.1186/s40359-023-01406-w (PMC10612317; doi:10.1186/s40359-023-01406-w)
Supplement: Supplementary file 1 — Supplementary Material 1 [file 40359_2023_1406_MOESM1_ESM.docx]

**SUPPLEMENTARY FILE 1**

**SEMI-STRUCTURED INTERVIEW GUIDE**

**Section A**

**Demographic Information Child**

1. Age…..

2. Sex…...

1. Education level…..
2. Diagnosis…..
3. Stage at diagnosis…….
4. Years/months of diagnosis…..
5. Treatment on/received…..
6. Is the child insured?........

**Parent**

9. Age:……………

10. Sex:………………

1. Relationship with the child:………………
2. Number of children...............
3. Education…..
4. Marital status….

15. Occupation:………………………………….

16. Average family income per month (GHC)…..

17. Religion:…………………………….. …….

18. Tribe:………………………………………..

19. Family history of cancer……….If yes, who?...Type….

**Section B**

**Guiding Questions**

**Child’s cancer symptom recognition and appraisal by parent**

1. Can you please tell me how you noticed that something was wrong with your child?
   - Can you tell me when you first noticed that something was wrong with your child?
   - What did you think it was?
   - What did you do when you noticed that something was wrong with your child?
   - Did you inform anyone about it? If yes, who? Why that person?
   - When did you inform the person?
   - What did the person say?
   - Did you seek help somewhere? If yes, where and what kind of help did you seek?
2. Can you please tell me at what point you took your child to the hospital?
   - How long was it from the time you noticed that something was wrong with your child to the time that you took your child to the hospital?
   - Which hospital did you take your child to?
   - What happened at the hospital?
   - What did the doctor say?
   - Were you asked to do any lab investigations?
   - Were you referred to any other hospital?
   - If yes, how long did it take you to go to that hospital?
   - What happened when you went to this hospital?
   - Did you do any lab investigations again?
   - How were you informed of your child’s cancer diagnosis?
   - Who informed you about the diagnosis?
   - Was someone else with you when you were informed? If yes, who?
   - What did you do afterwards?
   - What did the health workers do?
   - Who did you tell? Why that person?
   - Can you please tell me what your thoughts and feelings were when you were told that your child had cancer?
   - What happened next?
   - Have those thoughts and feelings changed since then?

**Impact of Child’s Diagnosis on The Family’s Functions**

1. Can you please tell me how long you have been caring for your child at home?
   - How are you able to tell when your child is experiencing symptoms at home?
   - How are you able to tell whether the symptom is mild or severe?
   - What do you do when your child is experiencing symptoms?
   - How do you know what to do when your child is experiencing symptoms?
   - How do you manage your child’s symptoms at home?
   - Who helps you to manage your child’s symptoms at home?
   - Can you tell me about the people who support you in managing your child at home?
   - Are you sufficiently supported?
   - What are your views concerning the advice these people give you?
   - How has your support changed?
   - What challenges do you face in caring for your child at home?
   - Apart from the symptoms you have mentioned so far, are there other kinds of care you provide for your child in relation to the cancer?
     - Physical care
     - Adherence to medication
     - Side effects of treatment

**Coping Strategies of parents**

1. Can you please tell me how having a child with cancer has affected you?
   - How has having a child with cancer affected your family?
   - In what ways has caring for your child affected your work?
     - Employment status
     - Attendance to work
     - Concentration at work

- How has caring for your child affected you financially?
- How has caring for your child affected your relationship with your spouse?
- How has caring for your child affected your relationship with others? Family members and friends.
- Has caring for your child with cancer affected the care you give to your other children? If yes, how?
- How has the care you are providing for your child affected you physically?
  - Sleep deprivation
  - Fatigue
  - Stress
  - Personal grooming
  - Pain
  - loss of appetite
- Do you have support from anywhere or from someone? If yes, who and what kind of support do you receive?
- Can you tell me how you cope with caring for your child?
  - Personal adjustment
  - Family support
  - Social support
  - Are there any personal characteristics you think have made this any easier for you to deal with? Or more difficult?
  - Are there any life’s events you think have made this any easier for you to deal with? Or more difficult?
  - Can you tell me about the things that help you manage your child at home?
  - Can you tell me if there is something that the hospital or a staff in the hospital has done/can do or is doing that has been helpful in making you cope better? If yes, who and what has the person done or can do?
  - Can you tell me if there is something that someone outside the hospital has done/is doing/or can do that helps or will help you cope better? If yes, who? What can the person do?

1. Is there anything else concerning your experiences that you would want to share?

**Thank you for your time and cooperation.**
